# Targeting Intracellular Mycobacteria Using Nanosized Niosomes Loaded with Antibacterial Agents

**DOI:** 10.3390/nano11081984

**Published:** 2021-08-01

**Authors:** Yael Nicole Slavin, Kristina Ivanova, Wei-lun Tang, Tzanko Tzanov, Shyh-dar Li, Horacio Bach

**Affiliations:** 1 Division of Infectious Diseases, Faculty of Medicine, The University of British Columbia, Vancouver, BC V6H 3Z6, Canada; yaelnicoleslavin@gmail.com; 2 Grup de Biotecnologia Molecular i Industrial, Department of Chemical Engineering, Universitat Politècnica de Catalunya, 08222 Terrassa, Spain; kristina.ivanova@upc.edu (K.I.); tzanko.tzanov@upc.edu (T.T.); 3 Faculty of Pharmaceutical Sciences, The University of British Columbia, Vancouver, BC V6T 1Z3, Canada; weiluntang1216@gmail.com (W.-l.T.); shyh-dar.li@ubc.ca (S.-d.L.)

**Keywords:** niosome, antibiotic, mycobacteria, macrophage, nanoparticle, lignin

## Abstract

Background: Pathogenic intracellular mycobacteria are challenging to treat because of the waxy and complex cell wall characterizing the genus. Niosomes are vesicles with biomimetic cell membrane composition, which allow them to efficiently bind to the eukaryotic cells and deliver their cargo into the cytoplasm. The objective of this study was to develop a new platform based on niosomes loaded with antimicrobial agents to target intracellular mycobacteria. Nanoniosomes were fabricated and loaded with antibiotics and lignin–silver nanoparticles. The efficacy of these nanoniosomes was tested against the intracellular pathogen *Mycobacterium abscessus* used as a model of infection of human-derived macrophages (THP-1). The cytotoxicity and the immunological response of the agents were tested on THP-1 cells using 3-(4,5-dimethylthiazol-2-yl)-2,5-diphenyltetrazolium bromide (MTT) assay and the secretion of pro- and anti-inflammatory cytokines, respectively. Results: *M. abscessus* was susceptible to the nanoniosomes in infected THP-1 macrophages, suggesting that the nanoniosomes were internalized due to their fusion to the macrophage cellular membrane. Moreover, nanoniosomes showed no upregulation of pro-inflammatory cytokines when exposed to THP-1 macrophages. Conclusions: Nanoniosomes improved drug efficacy while decreasing toxicity and should be considered for further testing in the treatment of intracellular pathogenic mycobacteria or as a new platform for precise intracellular delivery of drugs.

## 1. Introduction

Cystic fibrosis (CF) is a fatal genetic disorder that affects the lungs primarily, as well as the pancreas, liver, gut, and reproductive system. This disease is prevalent in the Caucasian population, affecting 30,000 people in the United States alone, and follows an upward trend observing a patient increase of 100% since 1986 [1]. CF is commonly accompanied by progressive lung disease with severe respiratory inflammation leading to mucous build-up due to high pathogenic bacteria gathering in the lungs. Ultimately, this causes respiratory failure and is the leading cause of death amongst CF patients [2,3].

Nontuberculous mycobacteria (NTM) are a group of bacteria that can be detected in the sputum of patients with CF, non-CF bronchiectasis, and chronic obstructive pulmonary disease (COPD). These bacteria are exceptionally problematic due to their antibiotic resistance and mechanism of invasion and proliferation within macrophages, which are the main phagocytic cell types of the innate immune system responsible for clearing pathogens. The prominent and rapidly growing mycobacteria that are responsible for persistent and often drug-resistant lung infections are *Mycobacterium abscessus* [4,5,6,7,8,9,10,11].

There is no standardized treatment against NTM, and an extensive array of antibiotics are utilized, including clarithromycin, amikacin, tigecycline, and cefoxitin [3,4,11,12,13,14]. The antibiotics can be used individually or in combination. However, pathogenic mycobacteria are challenging to treat and eradicate because of their complex waxy cell walls providing a barrier to antibiotics. Examples of NTM are *M. abscessus* and *M. avium* complex, which complicate chronic lung diseases such as bronchiectasis. With the aging of the population, NTM pulmonary disease rates are increasing, and the incidence of NTM growth from pulmonary specimens in British Columbia (Canada) was estimated at 6.7/100,000 from 1990 to 2006 [15]. NTM is an emerging threat to health as antibiotic therapies against these pathogens are poorly tolerated due to side effects and ineffectiveness of the multidrug-resistant strains.

Vesicular systems, such as liposomes and niosomes, are currently a hot topic of investigation with a potential solution to the lack of standardized treatment. Vesicular systems have been used as target drug delivery systems due to their lower toxicity and higher effectiveness than the free drug at the same concentration [16,17]. Niosomes, like liposomes, contain a bilayer (cholesterol and a surfactant). Unlike liposomes, they tend to be more stable because they do not comprise phospholipids susceptible to oxidative degradation [17,18]. The lowered toxicity, improved efficacy, and low cost make the niosomes an attractive method of drug administration for NTM infection treatment.

Silver has been used in medicine since ancient times and described by Hippocrates of Kos (c.460–c.370 BC) [19]. Over the last decade, the use of silver nanoparticles (AgNPs) has emerged as a promising treatment of bacteria because of their high reactivity based on their large surface area compared to their volume. AgNPs have a size ranging from 1 to 100 nm. Their toxicity mechanism appears to kill bacteria by generating reactive oxygen species (ROS) either in the vicinity or inside the bacterial cell [20]. Previous studies from our lab and others showed AgNPs kill planktonic cells and bacteria developing within biofilms [21]. Ultimately, AgNPs have antibacterial activities comparable to antibiotics. Recently, the use of colloidal Ag has been reported as a companion treatment of CF, supporting the use of AgNPs for the control of bacteria [22]. However, these NPs have some drawbacks, such as complicated fabrication, low stability, toxicity upon accumulation, and the possibility for inducing resistance development [23]. To overcome these challenges and enhance the AgNPs antibacterial activity at lower concentrations, we have employed “green”, simple, and relatively fast methodologies for the synthesis of the AgNPs using enzymes [24] and biopolymers [25,26] as reducing and capping agents.

In this study, we formulated nanosized niosomes (nanoniosomes) and encapsulated two drugs currently used to treat NTM infection: rifabutin [27] and ciprofloxacin [3,12,13]. Furthermore, we tested a new formulation based on the encapsulation of lignin-capped AgNPs (L-AgNPs). Encapsulation was performed using the antibiotics both individually and in combination. Drug concentration and the optimal drug-to-cholesterol ratio were determined by high-performance liquid chromatography (HPLC). Minimum inhibitory concentration (MIC) of the free drugs was calculated against *M. abscessus*, whereas the cytotoxicity and immunological response were tested against the human-derived cell line of macrophages THP-1. Finally, these cells were infected with *M. abscessus* and treated with nanoniosomes to assess the intracellular survival of the pathogen. We conclude that antibiotic- and L-AgNP-encapsulated nanoniosomes (L-AgNP-NIO) could be used to treat infected human cells at a concentration lower than the MIC of the free drug.

## 2. Materials and Methods

### 2.1. Measurement of Antibacterial Agent Concentration

Rifabutin and ciprofloxacin were obtained from Selleckchem (Houston, TX, USA) and Miles Laboratories (Elkhart, IN, USA), respectively. Ciprofloxacin and rifabutin were dissolved in acetonitrile.

Calibration curves of both antibiotics were prepared using 1, 2, 4, 5, 7, and 10 μM concentrations. The HPLC method was developed on a Waters 2695 Separations Module connected to a Waters 996 Photodiode Array Detector (Waters Limited, Mississauga, ON, Canada). The column used for this assay was a C_18_ 5 μm 150 × 4.6 mm InertSustain (GL Sciences, Tokyo, Japan). The mobile phases used were solvent A: water containing 0.1% formic acid and solvent B: acetonitrile, and 0.1% formic acid. The separation of the compounds was maintained at 37 °C. For sample preparation, 25 μL of nanoniosomes were mixed with 25 μL of acetonitrile, sonicated for 5 min, vortexed, and centrifuged at 15,000× *g* for 5 min. The supernatant (10 μL) from each sample was collected and placed in an HPLC autosampler vial for injection, which was performed in triplicate. The flow rate was maintained at 1 mL/min, and the antibiotics were monitored at λ = 272 nm and λ = 278 nm for ciprofloxacin (RT = 6.6 min) and rifabutin (RT = 5.7 min), respectively. 

L-AgNPs were synthesized via a one-pot redox reaction using alkali lignin (low sulfonate content, Sigma-Aldrich, St. Louis, MO, USA) as a stabilizing and reducing agent as published [28]. Briefly, 20 mL of AgNO_3_ (2 mg/mL) were mixed with 30 mL of lignin aqueous solution (1% *w/v* prepared in Milli-Q water, pH 5.5). The reaction was performed at 60 °C for 72 h under constant stirring. The obtained NPs were centrifuged at 18,000× *g* for 40 min to remove the unreacted lignin and the free Ag ions and then re-suspended in mQ water. 

The reduction of Ag ions to L-AgNPs was confirmed by measuring the UV-Vis spectrum of the suspension using Infinite 200 Pro, Tecan (Männedorf, Switzerland Austria). The spectrum was collected in the range of 300–550 nm with 2 nm step size recording number. The average size, size distribution, and zeta potential of the L-AgNPs were measured using Zetasizer Nano ZS (Malvern Instruments Inc., Westborough, MA, USA). L-AgNPs were visualized by a high-resolution transmission electron microscope (HRTEM), operated at 200KeV, JEOL (Tokyo, Japan). A powder of L-AgNPs was dispersed in ethanol using a sonication bath for 15 min. A droplet of the solution was placed on the copper grid and dried at room temperature before insertion into the microscope.

### 2.2. Nanoniosome Preparation

Empty nanoniosomes were prepared by dissolving 40 mg of each cholesterol and Tween-80 in 1 mL ethanol. The cholesterol stock was heated at 60 °C to ensure no residue remained. Then, 592 µL of the Tween-80 stock was combined with 408 µL of the cholesterol stock (70:30 molar ratio) and precipitated into 3 mL of 300 mM (NH_4_)_2_SO_4_ at a flow rate of 15 mL using a NanoAssemblr (Precision NanoSystems Inc., Vancouver, BC, Canada). The sample was then placed in a Slide-A-Lyzer Dialysis cassette 10K MWCO (Thermo Fisher, Walthan, MA, USA) and dialyzed sequentially against 300 mM (NH_4_)_2_SO_4_ for 1 h, HBS buffer (150 mM NaCl, 20 mM HEPES, pH to 7.4) for 1 h, and fresh HBS for 16 h.

For the drug loading process, a range of drug/cholesterol ratios varying between 1:10 to 1:50 were tested to identify the most effective loading ratio. For the nanoniosome loading with antibiotics, an aqueous stock solution of 10 mg/mL was prepared for ciprofloxacin, whereas 5 mg/mL in 25% ddH_2_O and 75% DMSO was used to prepare rifabutin. The preformed nanoniosomes were then mixed either with one or both drug solutions at a range of drug/cholesterol ratios (1/10–1/50, *w/w*, and 1/1, *v/v*) and incubated at 37 °C for 1 h, followed by an ice bath for 5 min. Samples were then consecutively dialyzed against fresh HBS twice for 1 h each to remove the unencapsulated drug.

### 2.3. Nanoniosome Size and Cholesterol Concentration

Nanoniosome size and size distribution were determined using a particle analyzer (Zetasizer Nano ZS, Malvern Instruments Ltd., Malvern, UK). Cholesterol concentrations in the nanoniosomes were measured using the Wako Cholesterol E enzymatic assay kit (Wako Chemical USA, Richmond, VA, USA).

### 2.4. Calculation of Encapsulated Drug

The concentration of antibiotics in the nanoniosome was measured as detailed earlier. Encapsulation efficiencies were calculated by comparing the drug [D] to cholesterol [C] ratios before and after dialysis using the following equation: (([D]/[C])_post_/([D]/[C])_pre_) × 100.

The concentration of L-AgNPs loaded in the nanoniosomes (L-AgNP-NIO) was calculated after disruption with acetonitrile (1:1), as described earlier. A calibration curve of the L-AgNP-NIO was determined in a 4% SDS-PAGE (Figure S1) after loading known concentrations of the L-AgNPs, and the values were calculated by densitometry. As indicated earlier, the final concentration of the L-AgNP-NIO was determined after their lysis (Figure S1).

### 2.5. Strains, Cell Line, and Culture Conditions

*M. abscessus* (ATCC 19977T) was grown in 7H9 broth (BD Biosciences, Franklin Lakes, NJ, USA) supplemented with 1% glucose and 0.05% Tween-80 at 30 °C with no agitation. The human-derived monocytic cell line THP-1 (ATCC TIB-202) was cultured in RPMI supplemented with 5% fetal calf serum (FCS, Invitrogen, Walthan, MA, USA), 100 mM L-glutamine (Hyclone, Logan, UT, USA), 100 U/mL penicillin (Hyclone), and 100 μg/mL streptomycin (Hyclone) at 37 °C supplemented with 5% CO_2_. The murine macrophage cell line RAW 264.7 (ATCC TIB-71) was used to quantify DNA proliferation by measuring the incorporation of BrdU (Cell Signaling Technology, Danvers, MA, USA). RAW cells were cultured in DMEM (Sigma-Aldrich) supplemented with FCS, glutamine, and antibiotics as detailed earlier and incubated as performed for THP-1 cells.

### 2.6. Minimum Inhibitory Concentration Detection

Minimum inhibitory concentration (MIC) was determined using the broth microdilution method. The MIC determines the concentration at which cell growth is inhibited. Testing was performed in round-bottom 96-well microtiter plates with each well-containing bacteria at a final optical density of 0.05 at λ = 600 nm. The antibacterial activity of the antibiotics was determined using a range of concentrations of 5–100 µg/mL or a range of nanoniosome concentrations of 1–100 µg/mL in a final volume of 100 µL with 7H9 broth as detailed earlier. All tests were performed in triplicate. Plates were placed in a 30 °C shaker for 16 h. Turbidity and/or large pellets were determined to be bacterial growth and further confirmed by plating. To confirm the bacterial growth, 10 µL of the sample was grown on 7H9 plates (broth supplemented with 1.5% (*w/v*) agar) for 24–72 h at 30 °C, and colony-forming units (CFUs) were counted post-incubation. The drug concentration of wells that were clear and had no bacterial growth was determined to be the MIC. Experiments were performed in triplicate.

### 2.7. THP-1 Cell Infection

Before the infections, 2 × 10^5^ THP-1 cells were differentiated to macrophages in a flat-bottom 96-well plate by adding 100 ng/mL phorbol myristate acetate (PMA) in RPMI and left overnight. The next day, cells were gently washed (×3), and 100 µL of fresh media supplemented with nanoniosomes containing antibiotic concentrations at 20 and 40 µg/mL were added in triplicate to allow their binding and the delivery of the antibiotics to the cell. On the day of the infection, *M. abscessus* was added at a multiplicity of infection of 1 (1 bacterium: 1 macrophage) after opsonization with 10% human AB^+^ serum (Gibco, Walthan, MA, USA) for 30 min at room temperature. After 2 h, 100 µg/mL of amikacin was added along with new antibiotic-containing media to kill non-internalized bacteria [29]. At time zero and every 24 h for 3 days, samples were treated with 0.025% SDS, serially diluted, and plated on 7H9 broth supplemented with 10% oleic acid, albumin, dextrose, and catalase (BD), and 1.5% agar. After plating, plates were sealed and incubated at 30 °C for 72–96 h until colonies were visible for counting. Experiments were performed in triplicate.

### 2.8. Cytotoxicity and DNA Proliferation

The MTT colorimetric assay was performed to determine the cytotoxic effect on THP-1 cells [30]. THP-1 cells were grown in RPMI as described earlier and seeded into flat-bottom 96-well microplates with 5 × 10^4^ cells/well combined with 100 ng/mL PMA and left to adhere overnight at 37 °C supplemented with 5% CO_2_. The following day, media was replaced, and varying nanoniosome (0.5–50 µg/mL), free drug (1–500 µg/mL), or L-AgNP concentrations were added in triplicate. Negative control wells were left untreated and positive control wells were treated with 1 µL of Tween-20. The plates were then returned to incubate at 37 °C, 5% CO_2,_ for 24 h. Each well was treated with 10 µL of MTT (5 mg/mL in PBS) and incubated for 4 h at 37 °C supplemented with 5% CO_2_. To solubilize the formed formazan crystals, 100 µL of solubilization solution (20% *w/v* SDS in a 50% dimethylformamide solution, containing 2.5% acetic acid and 2.5% HCl 1 M) was added to each well, and the plate was incubated again for 16 h. The OD was then measured at 570 nm with the Epoch microplate spectrophotometer (BioTek Instruments, Winooski, VT, US). Cell viability is represented as the corrected OD value compared to the untreated negative control. The cytotoxicity of L-AgNPs was also tested with the murine macrophage RAW cell line following the same protocol as THP-1, except for the inclusion of PMA in the initial cell seeding.

### 2.9. Immunological Response

Adherent THP-1 cells were used to measure the immunological response of the L-AgNP-NIO according to published protocols [31]. The pro-inflammatory cytokines IL-6 and TNF-α and the anti-inflammatory IL-10 were measured in the supernatant of THP-1 cells treated with 1 μg/mL nanoniosomes using commercial kits (B&D). These concentrations were chosen based on the cytotoxicity results, and experiments were performed in triplicate.

### 2.10. Statistical Analysis

Results were statistically analyzed with a *t*-test (Prism 6.0, GraphPad), and *p* < 0.05 was considered significant.

## 3. Results

### 3.1. Nanoniosome Size, L-AgNP Fabrication, and Encapsulation Efficiency

**Figure 1:**
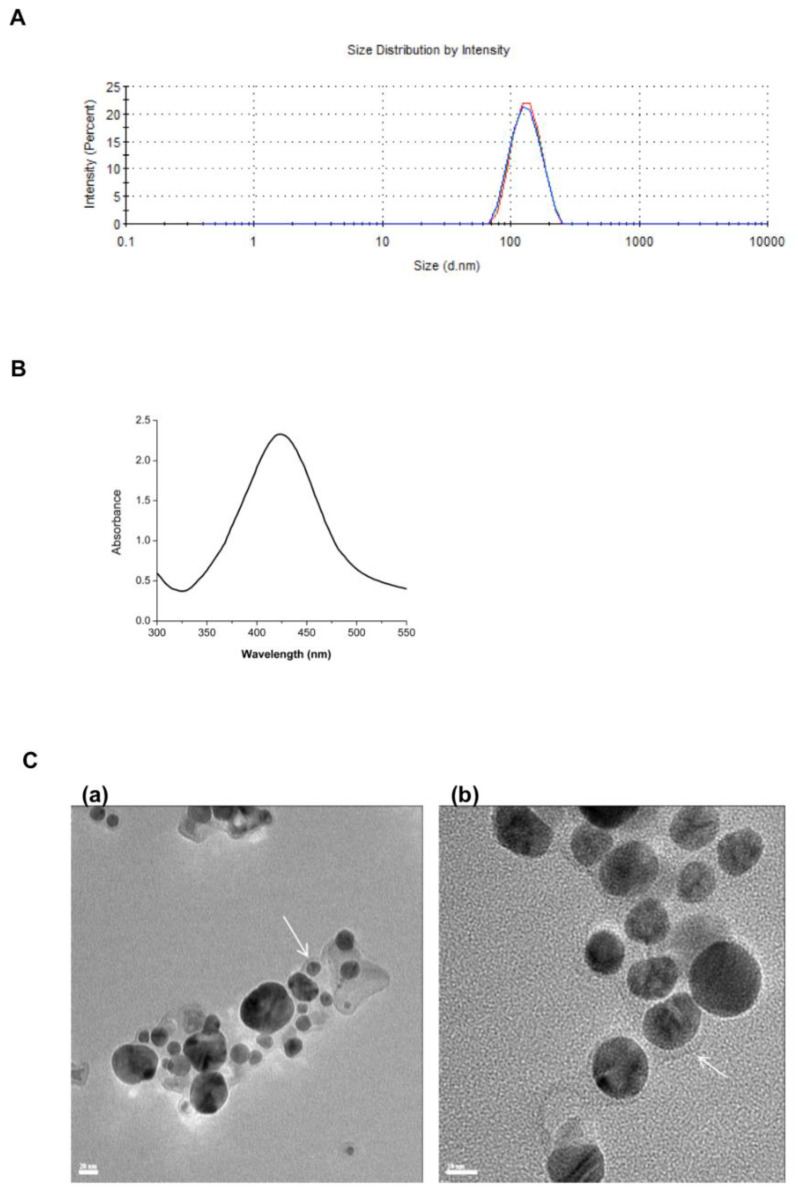
Characterization of L-AgNPs and nanoniosomes. The nanomaterials were characterized by (**A**) average size of the nanoniosomes by DLS, (**B**) UV-Vis spectrophotometry of the L-AgNPs, and (**C**) HRTEM of L-AgNPs with a scale bar of (**a**) 20 nm and (**b**) 10 nm. White arrows indicate the lignin layer. Measurements were performed in triplicate.

The size of the empty nanoniosomes showed an average diameter of 126.1 nm (Figure 1A) and a low polydispersity index of 0.053. UV-Vis spectrum with a characteristic absorbance peak at 420 nm confirmed the formation of the L-AgNPs (Figure 1B). The resulting L-AgNPs have an average particle size of 50.47 ± 1.05 nm and a polydispersity index of 0.38 ± 0.05, as was determined by dynamic light scattering. The zeta potential value of −45.45 ± 0.5 mV also confirmed the formation of stable NPs without a tendency to aggregate and precipitate. The morphology of the L-AgNPs was studied by HRTEM. The images showed L-AgNPs in a size of 10–20 nm homogeneously coated with the lignin layer, which showed a thickness of ~2 nm (Figure 1C).

**Figure 2:**
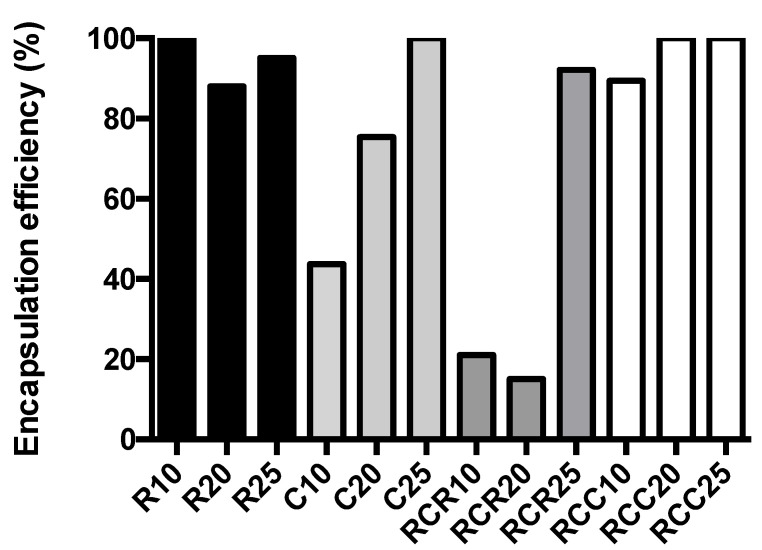
Encapsulation efficiency. Concentrations of encapsulated antibiotics were measured by HPLC using a C_18_ column according to Section 2.1. R = rifabutin, C = ciprofloxacin, RC = rifabutin and ciprofloxacin, RCC = concentration of ciprofloxacin combination, RCR = concentration of rifabutin in the combination. The numbers indicate the drug/cholesterol ratio.

Nanoniosomes were loaded with rifabutin (R-NIO), ciprofloxacin (C-NIO), or a combination of both antibiotics (CR-NIO) at ratios indicated in the methodology section. The drug-to-cholesterol ratio of 1:25 resulted in encapsulation efficiencies ranging from 92 to 100% (Figure 2) and thus was chosen as the ratio to prepare drug-loaded nanoniosomes. In the case of the L-AgNP-NIO, an encapsulation of 21% was calculated (Figure S1).

### 3.2. MICs and Cytotoxicity Results

The MIC for *M. abscessus* was 50 and 100 µg/mL when treated with rifabutin and ciprofloxacin alone, respectively. A minuscule pellet was observed at the 100 µg/mL treatment with ciprofloxacin. However, growth was not observed after plating and incubation, indicating that bacterial growth was indeed halted. *M. abscessus* was resistant to all nanoniosome treatments (with or without antibiotics) in an in vitro culture (data not shown), suggesting that the nanoniosomes were not able to interact with the cell wall of the pathogen. As expected, similar results were observed when L-AgNP-NIO were exposed to the pathogen in vitro (data not shown). However, exposure of the L-AgNPs alone effectively killed *M. abscessus* at a MIC of 5 µg/mL. The effectiveness of the L-AgNPs suggests that the mechanisms presented by NPs, as previously discussed, are more easily able to take place and attack the pathogen cell wall without an additional membrane from the nanoniosome acting as a barrier (since no fusion appears to occur) between the NPs and the pathogen.

**Figure 3:**
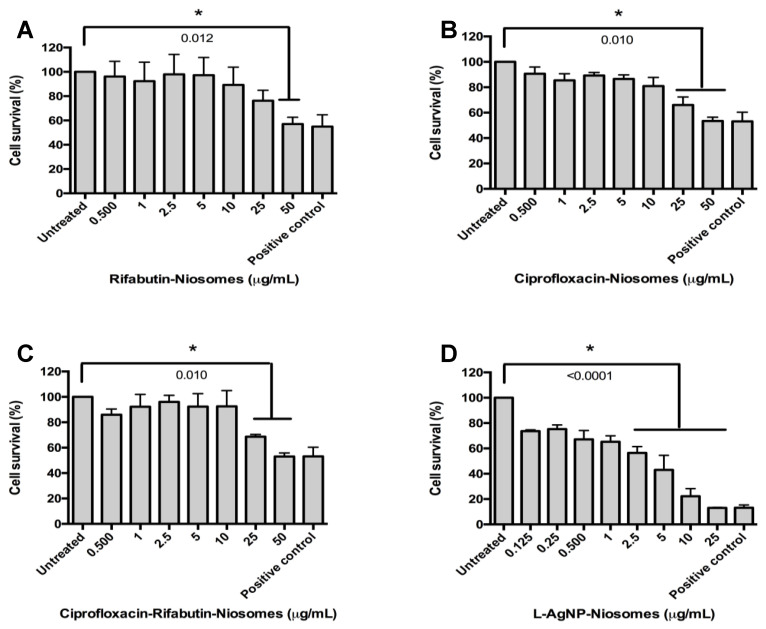
Cytotoxicity of nanoniosomes exposed to THP-1 cells. Antibiotic-loaded nanoniosomes were exposed to THP-1 cells, and the cytotoxicity was evaluated using the MTT assay. (**A**) R-NIO, (**B**) C-NIO, (**C**) CR-NIO, and (**D**) L-AgNP-NIO. Tween-20 and untreated cells were used as positive and negative controls, respectively. Shown is the mean ± SD of three independent experiments. * indicates the *p*-value < 0.05.

Cytotoxicity to THP-1 cells varied according to the treatment, and results are presented in Table 1 and Figure 3.

No cytotoxicity was measured amongst cells treated with empty nanoniosomes using the same volume as performed with antibiotic-loaded nanoniosomes. However, cytotoxicity of 2.5 µg/mL was measured when THP-1 cells were treated with L-AgNP-NIO. Deviation in the positive control in Figure 3D when compared to Figure 3A–C is due to data compression from statistical analysis. 

### 3.3. Nanoniosome Effect on Infected THP-1 Cells

**Figure 4:**
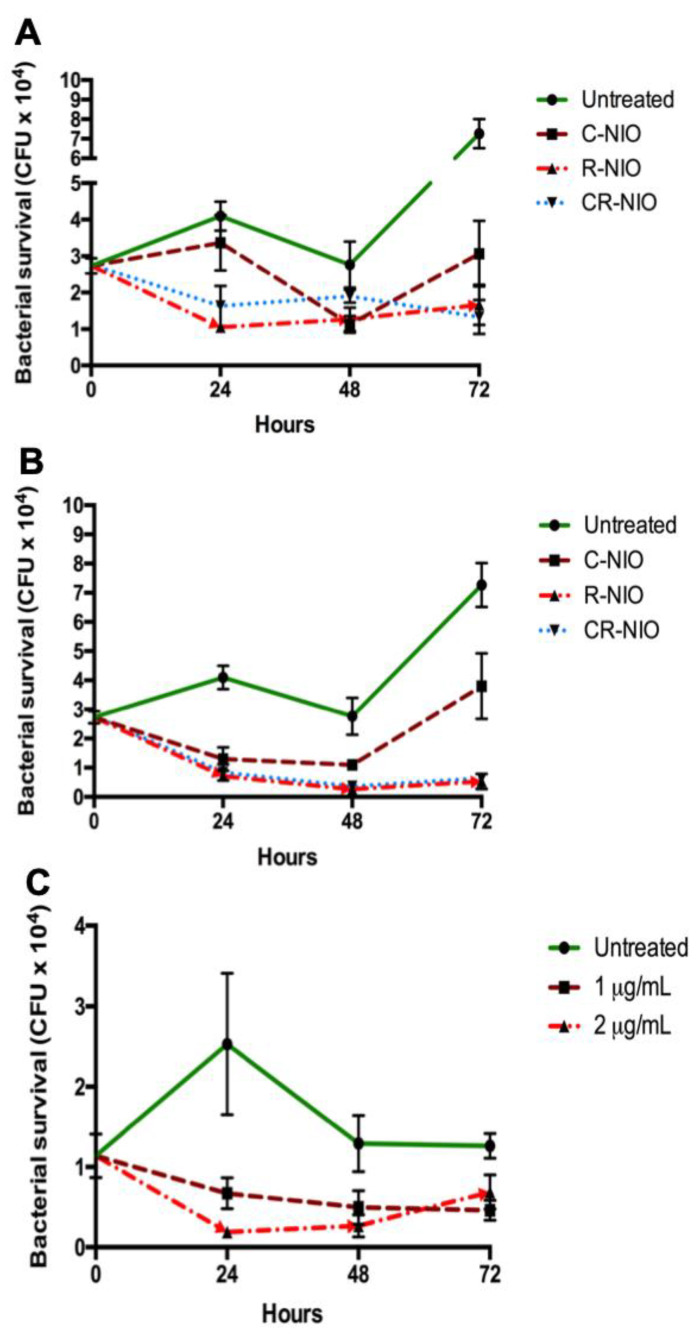
Growth of *M. abscessus* within THP-1 cells. THP-1 cells were infected with *M. abscessus* and exposed to nanoniosomes. The number of bacterial colonies was counted during THP-1 infection treated with nanoniosomes loaded with (**A**) 20 µg/mL and (**B**) 40 µg/mL antibiotics, and (**C**) L-AgNPs. CFU = colony-forming units. Shown is the mean ± SD of five independent experiments.

*M. abscessus*-infected THP-1 cells were plated throughout the experiment. C-NIO showed a weak antibacterial activity with 0.34 and 0.27 CFU log reductions when cells were treated with 20 and 40 µg/mL, respectively (Figure 4A,B). These concentrations were chosen as they fall below the MIC of the free drug, and nanoniosome encapsulation is known to enhance activity [32,33]. Higher antibacterial activities were measured when R-NIO and CR-NIO were tested with 0.80 and 0.83 CFU log reductions when concentrations of 20 µg/mL (Figure 4A) and 40 µg/mL (Figure 4B) were applied, respectively. However, the most effective treatments were the combination of both antibiotics CR-NIO at 40 µg/mL, which resulted in a 1.0 CFU log reduction of the bacteria, and surprisingly, R-NIO at 40 µg/mL resulted in a slightly stronger antibacterial activity with 1.1 CFU log reduction (Figure 4B).

When infected THP-1 cells were treated with 1 µg/mL of L-AgNP-NIO, a 0.83 CFU log reduction of the bacteria was measured after 24 h exposure. Interestingly, a slow recovery of the pathogen was observed in the following days (Figure 4C).

### 3.4. Inflammatory Response

**Figure 5:**
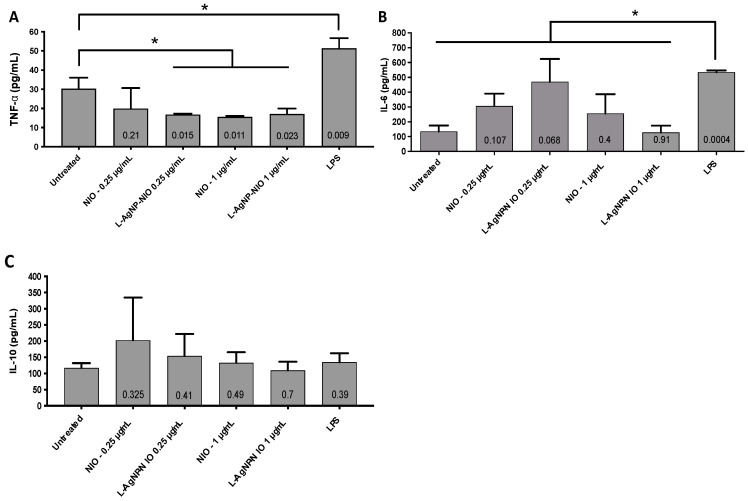
The immune response of THP-1 cells exposed to L-AgNPs and L-AgNP-NIOs. Adherent THP-1 cells were exposed to 0.25 and 1 μg/mL L-AgNPs (NIO) and L-AgNP-NIOs, and the secretion of the cytokines (**A**) TNF-α, (**B**) IL-6, and (**C**) IL-10 were measured in the supernatant. LPS = lipopolysaccharide. * is the *p*-value < 0.05. Shown is the mean ± SD of three independent experiments. Numbers in the bars indicate the *p*-value.

To determine whether an inflammatory response will be elicited due to exposure of THP-1 macrophages to L-AgNP-NIO, the secretion of the pro-inflammatory cytokines TNF-α and IL-6 were measured (Figure 5A,B). Results showed that no inflammation was recorded as the concentration of both cytokines was similar to the untreated cells. Similarly, when the anti-inflammatory activity of the L-AgNP-NIO was assessed with the secretion of IL-10, no increase in the secretion of IL-10 was measured (Figure 5C). Surprisingly, the effect of the L-AgNP-NIO at 0.25 and 1 µg/mL significantly decreased the level of TNF-α compared to the untreated control (Figure 5A), suggesting an anti-inflammatory response not linked to IL-10.

## 4. Discussion

In this study, we investigated a dual encapsulation of antibiotics in nanoniosomes. These nanoniosomes were tested for an intracellular delivery in *M. abscessus*-infected macrophages as a new antibiotic therapy.

As niosomes are currently emerging and still under development, studies differ significantly in their preparation. In our study, nanoniosomes were formulated with the non-ionic surfactant Tween-20 and cholesterol using a nanofluidization technique. This technique produced nanoniosomes of 126.1 nm, smaller than the majority of studies using alternative methodologies. Other studies reported the production of niosomes in a microsize range with variable encapsulation efficiencies. For example, niosomes encapsulated with ciprofloxacin using a transmembrane pH gradient produced sizes between 4.2 and 8.7 µm with encapsulation efficiencies ranging between 54% to 76% [17]. Another study used lipid layer hydration with resulting sizes between 12 and 36 µm and encapsulation efficiency of 80% [18]; however, β-cyclodextrin was used to improve the solubility of ciprofloxacin during antibiotic loading.

Moreover, other studies used a thin-film hydration methodology to encapsulate ciprofloxacin. In this regard, niosomes were produced in a range of 200–1200 nm, but afterward, the microsized vesicles (multilamellar) were sonicated to reduce their size. Surprisingly, niosomes after sonication showed a wide range of sizes as suggested by the polydispersity index between 0.3 and 0.8 [32,33]. In addition, the encapsulation of other antibiotics such as clarithromycin using the same method produced niosomes with an average size of 4.67 µm and a polydispersity index varying between 0.23 and 0.72. In conclusion, in the present study, we report that a homogeneous population of nanoniosomes was achieved with a very low polydispersity index of 0.053.

We found that a high encapsulation (around 100%) was obtained regarding the encapsulation efficiencies when the ratio between antibiotic-to-cholesterol was 1:25 for both antibiotics. Two existing studies have successfully shown simultaneous encapsulation with the antimycobacterial drugs rifabutin and isoniazid [34,35]. Although the drug delivery was successful in both studies, the resulting particles were microsized, whereas the niosomes reported here were nanosized.

To determine whether or not the dual encapsulation of antibiotics effectively treats intracellular microorganisms, we chose *M. abscessus* as an intracellular model of infection in macrophages. The pathogen was resistant to all encapsulated drugs (no MIC detected) when tested using the broth microdilution method. This indicates that the nanoniosome formulation did not release the antibiotic, suggesting that no fusion of the nanoniosomes with the bacterial cell wall caused the antibiotic delivery. This fact is supported by the different architecture of the bacterial cell wall compared to eukaryotic membrane cells. In addition, bacteria lack cholesterol, making it difficult to fuse the niosomes with their cell wall [14,36].

*M. abscessus* is an intracellular pathogen that can infect macrophages [36]. This bacterium can commonly be found in lung infections and those prone to lung infections, such as CF patients [3]. Results of infected macrophages treated with nanoniosomes showed that they could fuse to the membrane and deliver their cargo in macrophage cells, compensating for the inefficacy of the free drug [32]. Our experimental model observed bacterial growth within macrophage cells over 72 h and showed that C-NIO did not show a strong antibacterial effect nor a synergistic effect when paired with rifabutin. This contradicts other findings that showed significant antibacterial activity exhibited by ciprofloxacin niosomes towards *Francisella tularensis* [37], *Staphylococcus aureus*, and *Escherichia coli* [32]. However, niosome efficacy is found to be higher in Gram-negative bacteria [32], leading to the hypothesis that decreased C-NIO bactericidal activity in this study could be due to the unique mycobacterial wall that establishes an intrinsic resistance. Additionally, because ciprofloxacin was shown to have a 2x higher MIC than rifabutin, in the case of membrane fusion drug levels may not have been enough to cause cell damage with C-NIO as compared to R-NIO. It is also possible that daily doses of nanoniosomal treatments would lead to complete inhibition of bacterial growth as a minor decrease in growth was detected with C- and CR-NIO.

Exposure of the L-AgNPs to the pathogen resulted in a MIC of 5 µg/mL, which is an expected value compared to other reports where AgNPs were used [31,38,39,40]. The use of lignin during the fabrication of the AgNPs was chosen because of its high biocompatibility with cells [41] and the fact that the presence of hydroxyl groups (pH 10.5 according to Sigma documentation) favored the loading of the nanoniosomes because of the gradient difference during loading [42].

Liposomal-encapsulated ciprofloxacin has been shown to exhibit antibacterial activity [43]. However, it has also been shown that the drug does not remain within the target tissues, limiting the possibilities of medical application [44]. Liposomal-encapsulated rifabutin has also demonstrated antibacterial activity [45]. Again, inconsistencies can be due to fusion issues as liposomes consist of phospholipids in place of a niosomal nonionic surfactant.

In contrast to C-NIO, R-NIO at 40 µg/mL exhibited an intracellular antibacterial effect that resulted in a 1.1 log reduction over 3 days using only one dose; a result that was similar but more bactericidal compared to the 1 log reduction obtained by CR-NIO at 40 µg/mL. This shows that ciprofloxacin has a negligible activity when encapsulated with rifabutin to treat intracellular infection, and no synergistic effect occurs between the two drugs. Nevertheless, 40 µg/mL is a concentration below MIC, indicating that there may be an increased targeting mechanism in play leading to lower drug concentrations having an amplified effect. Rifabutin shows efficacy in treating *M. abscessus* infections [27] and should be revisited as a potential treatment for CF and lung diseases in the future. 

The fact that intracellular ciprofloxacin was not efficient in reducing the load of *M. abscessus* has already been reported for *Legionella monocytogenes* [46]. In this study, the authors reported an accumulation of fluoroquinolones in macrophages and decreased intracellular activity of ciprofloxacin against the pathogen compared to the results obtained extracellularly. One of the explanations provided by the authors was the thick layer of actin that surrounds the cell wall of the bacteria, reducing the effect of the antibiotic. Other explanations are based on the distribution and compartmentalization of ciprofloxacin in eukaryotic cells. In this regard, most accumulated ciprofloxacin in macrophages occurs in the cytosol [47], whereas the natural habitat of *M. abscessus* in macrophages is in the phagosome. Moreover, acidified compartments in the macrophage, such as the phagosome (pH ~5.5), significantly decreased the antibacterial activity of ciprofloxacin [48].

When the cytotoxicity of the antibiotics and the nanoniosomes was assessed in macrophages, rifabutin alone showed high toxicity to THP-1 cells (1 µg/mL), encapsulated the nanoniosome formulation, and the antibiotic was more tolerable to the THP-1 cells at 50 µg/mL. In contrast, the nanoniosome formulation made ciprofloxacin more harmful to the THP-1 cells with a cytotoxicity level at 25 µg/mL, while free drug cytotoxicity was 500 µg/mL. Encapsulation has been shown to decrease cytotoxicity [17,32], but the discrepancies amongst results may be due to a combination of the fact that both drugs can infiltrate macrophages [14,36]. Different kinetics of internalization lead to varying bactericidal activity, altering the usual drug mechanism of action. Other studies have shown free drug toxicity at a range from 0.004 µg/mL [34] to complete resistance [34]. This range is so extensive because the studies treat diverse bacterial strains exhibit different levels of intrinsic and acquired resistance. Mycobacteria specifically have innate and acquired resistance [4,10,13,49]. However, the viability of the macrophages reported in our study (Table 1) is in the range of 70%, which has been determined to be at a safe limit [50].

Exposure of the L-AgNP-NIO to THP-1 cells did not induce any inflammatory activity. However, a significant decrease of the TNFα in the supernatant was observed. The reduction of the level of this cytokine could be the result of the presence of phenolic groups in the lignin molecule. Lignin is composed of derivatives of phenylpropane. Although lignin per se has not been demonstrated to have any activity in the regulation of TNFα, some natural compounds based on phenylpropane have shown that effect. For instance, the phenylpropane sandaracopimaradiene obtained from the plant *Amentotaxus formosana* was shown to decrease the secretion of TNFα in the microglia cell line N9 [51]. Similar results were reported with anethole, another phenylpropane derivative from natural sources, in a periodontitis model [52].

There have been no previous studies examining the inflammatory effect of a nanoniosome. Still, AgNPs alone have been found to modulate the expression levels of the cytokines we have tested: IL-6, IL-10, and TNFα. For example, increases in the secretion of both pro-inflammatory cytokines IL-6 and TNFα were reported when AgNPs were exposed to murine and human macrophages [38,53,54]. The same trend of increase in the secretion of the anti-inflammatory IL-10 has been reported in both models [38,53,54]. While not explicitly tested with macrophage-like cells, niosomes alone have been found to both elicit anti-inflammatory effects [53] as well as the increase of the cytokines IL-2 and interferon-γ [54].

In conclusion, in this study, nanoniosomes loaded with antibiotics and biopolymer-metal NPs are reported. Results showed that nanoniosomes were not effective in vitro when exposed to the pathogen *M. abscessus* but exhibited antibacterial activity in *M. abscessus*-infected macrophages. Our study shows that the rifabutin drug delivery system can target intracellular infections while improving efficacy and safety, confirming that nanoniosomes could potentially be used to treat *M. abscessus*, a notoriously resistant bacterial complex. In addition, although ciprofloxacin showed a weak antibacterial activity, the dual encapsulation of antibiotics in nanosized vesicles is possible, opening a new field of therapeutics using different antibiotic combinations rather than rifabutin–ciprofloxacin.

## Figures and Tables

**Table 1:** Toxicity of the different treatments in THP-1 cells.

Treatment	Cytotoxicity (µg/mL)	Cell Viability (%)
Rifabutin	1	76.29
Ciprofloxacin	500 (>250)	68.37
R-NIO	50	74.87
C-NIO	25	77.91
CR-NIO	25	78.81
L-AgNPs	25	55.29

## Data Availability

Data are available upon request.

## Supplementary Materials

The following are available online at https://www.mdpi.com/article/10.3390/nano11081984/s1, Figure S1: The concentration of L-AgNPs loaded in the nanonisomes was calculated after disruption with acetonitrile (1:1). (A) A calibration curve of the L-AgNPs was determined in a 4% SDS-PAGE after loading known concentrations of the L-AgNP, and the values were calculated by densitometry. (B) The final concentration of the L-AgNPs in the nanoniosome was determined after their lysis, as indicated earlier. The black arrow points to the released L-AgNPs.
