# Supplementary material for: Targeting Intracellular Mycobacteria Using Nanosized Niosomes Loaded with Antibacterial Agents
Source: Nanomaterials (Basel). 2021 Aug 1;11(8):1984. doi: 10.3390/nano11081984 (PMC8398725; doi:10.3390/nano11081984)
Supplement: Supplementary file 1 [file nanomaterials-11-01984-s001.zip › nanomaterials-1285834-supplementary.pdf]

Supplementary Information

Targeting Intracellular Mycobacteria Using Nanosized Niosomes Loaded with Antibacterial Agents

Yael Nicole Slavin [1], Kristina Ivanova [2], Wei-lun Tang [3], Tzanko Tzanov [2], Shyh-dar Li [3] and Horacio Bach [1,*]

[1] Division of Infectious Diseases, Faculty of Medicine, The University of British Columbia, Vancouver, BC V6H 3Z6, Canada; yaelnicoleslavin@gmail.com
[2] Grup de Biotecnologia Molecular i Industrial, Department of Chemical Engineering, Universitat Politècnica de Catalunya, 08222 Terrassa, Spain; kristina.ivanova@upc.edu (K.I.); tzanko.tzanov@upc.edu (T.T.)
[3] Faculty of Pharmaceutical Sciences, The University of British Columbia, Vancouver, BC V6T 1Z3, Canada; weiluntang1216@gmail.com (W.-l.T.); shyh-dar.li@ubc.ca (S.-d.L.)
* Correspondence: hbach@mail.ubc.ca; Tel.: +1-604-875-4111

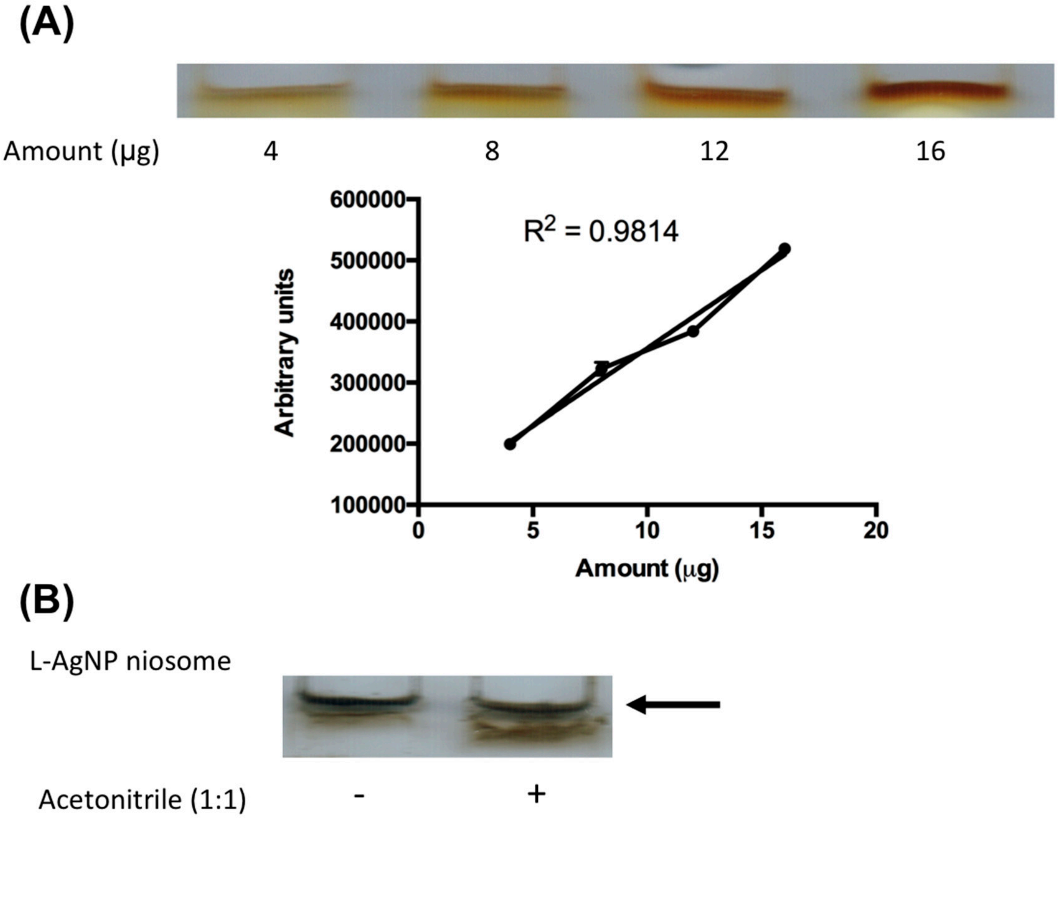

Figure S1. The concentration of L-AgNPs loaded in the nanonisomes was calculated after disruption with acetonitrile (1:1). (**A**) A calibration curve of the L-AgNPs was determined in a 4% SDS-PAGE after loading known concentrations of the L-AgNP, and the values were calculated by densitometry. (**B**) The final concentration of the L-AgNPs in the nanoniosome was determined after lysis, as indicated earlier. The black arrow points to the released L-AgNPs.
